# London Correspondence

**Published:** 1859-05

**Authors:** 


### *London Correspondence.*

MESSRS. EDITORS: Health is found here, as it is elsewhere, to be improved, and life to be prolonged, as sanitary measures are more extensively carried out; and to this end some of the first medical talent has been directed for years, aided by minds devoted to collateral branches of science.

The result has been a greatly diminished mortality, not only in London, but throughout the Kingdom. The death-rate for the year which has just closed, notwithstanding the great mortality caused in all parts of England in the last quarter of 1858, by diptheria, was less than for many previous years; in short, two less in every thousand than for ten antecedent years—being a saving of two hundred and fifty lives to the country in one year. It is a novel sight, when new sewers are being made, or old ones opened, to see how they intersect each other at distances from *ten* to *forty* feet below the surface; and at times, should curiosity prompt, one may walk for miles under the heart of this great and modern Babylon, in one of these underground chambers lighted with gas, the walls of the largest, I believe, being between four and five feet thick, and the tube twelve or fourteen feet in diameter, or capacious enough, you may fancy, to carry a large quantity of refuse into the Thames.

That the water of this river may no longer be contaminated, and send forth from its banks (as was the case during the unusual heat of last summer) a noisome stench, the sewerage of London is to be carried about thirty miles below the city before being emptied into the stream. It is a great undertaking, and the outlay very large, say about six millions of pounds. Last summer, too, the odour from the serpentine in Hyde Park, led to much discussion, and as the plentiful supply of lime, which was daily cast in at an expense of much over one thousand pounds per day, produced little effect above killing all the fishes, the water of the serpentine will most likely be drawn off entirely, the bottom cemented, flood-gates placed at one end, and the water allowed to escape as occasion may require, for the purpose of a fresh supply.

You may now like a sweeter subject than sewerage, and a more lively one than can be found in death-rate statistics; and this brings me to the subject of your request made so long ago, i. e. that I should send you something for your Journal. Without making this after the manner of a special communication for that purpose, you may be able, perhaps, to select some portion, and by giving to it a *name* and a *place*, appropriate it to your use. Beyond the range of medical science, there are many subjects of national, political and general interest, to absorb the attention in this busy, restless metropolis, whose great heart swells by night and by day at the sound of "merry meetings and delightful measures" of some of the millions of this ever onward moving mass. Whilst with others, alas! who find no home but a prison, no shelter but a refuge, no covering but tattered rags, no food but dry bread, to protract a little longer a living death, the heart indeed may beat and swell, but it will only be with the anguish of bitter despair. And yet the amount of money given to relieve the poor is enormous. There are some night refuges in London, where shelter is given to a limited number (i. e. from one hundred to six hundred in each house of Refuge) of men, women and boys for the night, with a plentiful supply of water and accommodation for warm and cold baths, each person who enters (unless there is some substantial reason for departure from the rule) being required to take a bath twice a week. In addition to the protection for the night, the males are supplied with eight ounces of dry bread, night and morning, and the females the same, with coffee. I have lately visited some of these places, upon which so much has lately been written, and in response to an article in the "Times," of December last, up to the present time, about £13,000, in small and large sums, have been given in aid of the funds.

Have you any idea of crossing the Atlantic next summer? If so, I need not repeat that I shall expect the pleasure of seeing you. I very frequently visit the Hospitals, where so much of interest is to be seen; on another occasion I will send you some cases.

The "Obstetrical Society of London," of which I am to become a member, has just started vigorously into existence.

Dr. Rigby is the President, and we may presume that all the new births which the Society introduces into "this breathing world" for the current year, will be perfectly *legitimate!* At the second meeting which was held a few evenings ago, a very elaborate paper from Dr. Tyler Smith was read, the main feature of the paper being a strong protest against craniotomy; indeed, Dr. Smith would discard such a measure altogether from his obsterical practice. How then would he avoid the desideratum in cases of imminent danger to the mother?—By denying, if I rightly understood the argument, the necessity for the existence of such a state of, or conjuncture of affairs, as would, under the generally adopted principles of practice, lead to the propriety, or the necessity of such an operation. To this end, Dr. Smith urges the propriety, and the salutary effect of the adoption of a digital examination some months before the period of the expected labor, in order that, should mal-formation, or deformity, or any extraneous cause exist in the parturient woman, that would operate as a bar to her safe delivery, or that would endanger the life of the child at the full time, in such cases abortion should be produced. Of the mode of effecting this, Dr. Smith has the concurrent testimony of obstetricians of equal note with himself. I allude to the injection of warm water into the uterus. The moral and religious grounds which have been urged against such procedure are easily disposed of. What the safety of the mother demands is omnipotent.

But for the late hour at which Dr. Smith's paper was brought to a close (another having been read before) much discussion, upon some of the views maintained by the author, would have been elicited.

At the close of the year all the papers read before the Society are to form a handsome volume, when you may have an opportunity of judging of the zeal and energy of many of the members of the "Obstetrical Society of London."

Extending one's remarks upon women a little further, I may mention, *en passant*, that Mr. Baker Brown, of St. Mary's Hospital, has just resigned the post which he has so successfully filled for some years. Who will be the new aspirant for fame in relieving the fearful maladies of the sex, to which Mr. Brown has devoted so much time and attention, is not at present known. The button suture of Doctor Bozeman is held in much favor, and is frequently employed at some of the Metropolitan Hospitals, with good, not to so say universal, success.

The late President of the Harveian Society, Dr. Hamilton Roe, retired from the chair in a very satisfactory manner to himself and to the Fellows of the Society, by giving at his house a *conversazione*, where microscopical examinations of the solids and fluids which enter into the composition of that paragon of animals—man—passed in pleasant review, and all things needful to some few hours pleasantly occupied brought the evening to a close.

I will now for a short time revert to Surgery—which, in London, as everywhere, at the present day, is eminently conservative, and, referring to one branch, I may say that surgeons generally *crush* for stone, having due regard to the state of the bladder, prostate gland and urethra—to-wit: the bladder of sufficient capacity to retain five or six ounces of water in a tolerably quiescent state, the gland not *much* enlarged and the urethra not irritable. Under these circumstances (and even when some of them were in a degree infringed upon), I have frequently seen Mr. Coulson operate upon private patients with complete success—five or six sittings, of as many, or of ten minute's duration, without chloroform, being required to complete the operation. The age of one person from the country was seventy, five. Much practice is required to grasp the stone in the lithotrite. and great caution to grasp nothing *but* the stone. Mr. Ferguson, of King's College Hospital, frequently performs the operation also.

You may have seen something of Mr. Holt's, of the Westminister Hospital, operation for stricture. I am inclined to believe that it does not find very general favor with the profession, and so far as the idea of the manner is concerned, not much with the patients themselves.— It consists in forcibly, at one trial, *splitting up* the strictured part of the urethra, after this introducing and withdrawing a No. 10 sound, then sending the patient to bed and occasionally, during three weeks, passing an instrument.

The superiority claimed by Mr. Holt for this mode of operating is, that the stricture does not return. About two months ago Mr. Holt told me that he had performed his operation eighty-four times, only one being a failure—a much greater success certainly than can be claimed by those who descend by slow, perhaps easier gradation, the delicate pathway of a male urethra.

I do not remember, whilst a resident of the Crescent City, to have met with a medical friend who had employed a solution of morphine sub-cutaneously in cases of delirium tremens. A short time ago, Dr. Fuller, of St. George's Hospital, reported, at a medical meeting, one case, amongst others, in which he had injected half a grain of morphine in half a drachm of water, with almost immediate effect—this case having resisted the action of many previous remedies. The sleep produced was of twelve or fifteen hour's duration, the patient arousing from it, well.

The apprehended results in cases treated in this way, of fatal narcotism, have not been verified. The part chosen for the injection is not insisted on, avoiding, of course, close proximity to large blood vessels—the arm is, however, usually selected. A capital little instrument has been adapted to the purpose by a surgical instrument maker in St. James st., being a minute glass syringe with an exceedingly fine point to be inserted into the slight incision which has been made into the skin. If, in the unfortunately frequent opportunities which occur in New Orleans, a trial of this plan of treatment should be carried into effect, I shall be happy to hear the result, and equally so to receive your Journal which you kindly offered to send.

The winter here has been remarkably mild; scarcely any cold weather since the early part of November; indeed such a winter as would not affect the susceptibility of many tender exotics. The last was widely different.

It has just occurred to me that I might say a few words upon one other subject. The frequent cases of death which occur from the sale of poisons by chemists, has claimed the attention of Parliament, and very soon a bill is to be brought in by Mr. Walpole, regulating the sales of poisons and the manner of keeping them in the shops.

In a former bill twenty-three articles were enumerated; now the number coming within the provisions of the act is reduced to thirteen. The bill is framed with a view to the public good, and at a future time it may be well to examine closely into it. The recent wholesale poisoning, at Bradford, by the sale of arsenic, in mistake for an article called daff, (sulphate of lime) which is used to adulterate candy, has brought the subject forcibly before the public. B.

[*N. O. Med. News & Gaz.*

